# Tumor-Infiltrating Lymphoid Cells in Colorectal Cancer Patients with Varying Disease Stages and Microsatellite Instability-High/Stable Tumors

**DOI:** 10.3390/vaccines9010064

**Published:** 2021-01-19

**Authors:** Salman M. Toor, Varun Sasidharan Nair, Khaled Murshed, Mohamed Abu Nada, Eyad Elkord

**Affiliations:** 1 Cancer Research Center, Qatar Biomedical Research Institute (QBRI), Hamad Bin Khalifa University (HBKU), Qatar Foundation (QF), Doha 34110, Qatar; mstoor@hbku.edu.qa (S.M.T.); vsnair@hbku.edu.qa (V.S.N.); 2 Department of Pathology, Hamad Medical Corporation, Doha 34110, Qatar; kmurshed@hamad.qa; 3 Department of Surgery, Hamad Medical Corporation, Doha 34110, Qatar; mabunada@hamad.qa; 4 Biomedical Research Center, School of Science, Engineering and Environment, University of Salford, Manchester M5 4WT, UK

**Keywords:** colorectal cancer, T cells, B cells, NK cells, immune checkpoints, dMMR, MSI-H, MSS

## Abstract

Immune checkpoint inhibition is an effective anti-cancer therapeutic approach but has shown limited efficacy in treating colorectal cancer (CRC) patients. Importantly, immune constituents of the tumor microenvironment (TME) can influence therapy response and cancer progression. We investigated the expression of immune checkpoints (ICs) on lymphoid populations within the CRC TME and compared with cells from normal colon tissues using samples from 50 patients with varying disease stages. We found that the levels of B cells, T cells, and NK cells were similar, IC-expressing CD4^+^ and CD4^+^CD8^+^ double positive T cells were higher, while CD8^+^ T cells and CD4^−^CD8^−^ double negative T cells were significantly lower in CRC tumors. Notably, patients with mismatch-repair deficiency/microsatellite instability-high tumors had higher levels of IC-expressing CD4^+^ and CD8^+^ T cells than patients with proficient MMR and microsatellite stable tumors. Lastly, The Cancer Genome Atlas Colon Adenocarcinoma datasets showed associations between low expression of selective genes and poorer progression-free interval. Our findings highlight differential expression of ICs on lymphoid cells in CRC tumors in the era of cancer immunotherapy, which at present is solely approved for anti-PD-1 therapy in patients with dMMR/MSI-H tumors. Further investigations into their functionality have potentials for deciphering resistance mechanisms to IC inhibition.

## 1. Introduction

Colorectal cancer (CRC) is the third most frequently diagnosed cancer amongst both sexes, accounting for 9.2% global cancer-related mortality [1]. Promising anti-cancer therapies such as immunotherapy have shown great advances in recent years but warrant additional insights into interactions between tumor cells and tumor-infiltrating immune cells (TIICs) to overcome resistance mechanisms and to improve response to therapy.

Immune constituents of the tumor microenvironment (TME) can influence disease initiation, affect rate-limiting factors for disease progression and ultimately impact disease prognosis [2]. Cells of myeloid and lymphoid lineage within the TME have both anti- and pro-tumor roles, which may suppress tumor growth via production of pro-inflammatory cytokines or direct tumor cell killing, or support tumor progression via angiogenesis and invasion by favoring cell proliferation [3]. Correlations between immune infiltrates and disease prognosis have been widely reported in different cancers including CRC [4]. Importantly, high levels of Th1/cytotoxic memory T cells in the tumor core and at the invasive margin are associated with prolonged disease-free survival (DFS) and overall survival (OS), accompanied with reduced threat of relapse and metastatic disease in CRC patients [5,6]. Therefore, lymphoid populations within the TME have cardinal roles in tumor progression and in orchestrating response to therapy.

Immune checkpoints (ICs) have emerged as vital targets for eliciting potent anti-tumor immune responses, primarily via inactivation of inhibitory immune receptors within the TME [7]. Accumulating evidences have shown upregulation of ICs and their ligands within the TME across different malignancies [8]. Studies have disclosed that IC pathways have critical roles in the pathogenesis of CRC as evident from single nucleotide polymorphisms of important IC genes in CRC patients [9], accumulation of IC-expressing immune cells in the TME [10] and associations with TIL load, rate of mutations, and survival [11]. Therefore, blocking IC pathways for therapeutic benefits has been comprehensively explored in treating CRC patients.

Studies have shown that CRC patients presenting with mismatch-repair deficiency (dMMR) and microsatellite instability-high (MSI-H) tumors exhibit sustained and durable clinical responses to IC blockade, compared to patients with proficient MMR [12]. Loss of MMR proteins provide phenotypic evidence of MSI-H tumors with dMMR, whereas MMR-proficient tumors comprise MSI-low or microsatellite stable (MSS) tumors with unmarked MMR proteins [13]. However, the favorable effects of MSI-H tumors on successful IC inhibition are often decreased in advanced stages or in metastatic CRC [14]. Moreover, overall response rates among all CRC patients treated with IC inhibition remain low, primarily due to tumor intrinsic/extrinsic mechanisms of resistance or recurrent accounts of immune-related adverse events (irAEs) [15,16]. In addition, evolving ICs such as T cell immunoglobulin and mucin domain-containing protein 3 (TIM-3), lymphocyte activation gene 3 (LAG-3), T cell immunoreceptor with Ig and ITIM domains (TIGIT) and inducible T cell costimulatory (ICOS) among others, are relatively in adolescent phase in their clinical translation as part of cancer immunotherapy regimens, compared to established ICs; programmed cell death protein 1 (PD-1) and cytotoxic T-lymphocyte-associated protein 4 (CTLA-4).

In this study, we performed critical immune cell profiling of colorectal tumors by focusing on lymphoid cell populations within the TME and investigated expression levels of ICs in a CRC cohort comprising of patients with varying disease stages. We investigated the levels of tumor-infiltrating B cells, T cells and NK cells compared to normal tissue milieu and established the expression profiles of PD-1, TIM-3, LAG-3, TIGIT, and ICOS on the different tumor-resident lymphoid populations. We also investigated associations between the levels of different IC-expressing lymphoid cells in the TME and disease stage to decipher their plausible roles in tumor progression. Importantly, we identified patients with MSI-H and MSS from our cohort and compared the levels of tumor-infiltrating lymphoid cells and IC expression between them. Lastly, we explored the clinical importance of our findings by investigating gene expression levels of the selected markers that have been elevated in CRC tumors with patient survival data from The Cancer Genome Atlas Colon Adenocarcinoma (TCGA-COAD) datasets.

At present, only anti-PD-1 is approved for treating CRC patients with unresectable or metastatic dMMR/MSI-H tumors, and only recently approved as a first-line treatment in these patients, based on KEYNOTE-177 study [17,18]. Other IC pathways are currently being explored for therapeutic benefits in CRC patients. Therefore, our main focus was to provide insights into the expression levels of different IC molecules in CRC tumors, while highlighting the significance of dMMR/MSI-H tumors. Our data highlight the role of different lymphoid cells within CRC TME and uncover the expression levels of emerging ICs on different populations within the TME, which is supported with patient survival data from TCGA datasets, to provide credible targets for successful IC inhibition for improving clinical outcomes in CRC patients.

## 2. Materials and Methods

### 2.1. Sample Acquisition and Storage

This study was approved by the Institutional Review Boards at Qatar Biomedical Research Institute (QBRI) (Study no. 2018-018) and Hamad Medical Corporation (HMC) (Study no. MRC-02-18-012), Doha, Qatar.

Tumor tissues (TT) and corresponding, non-tumor normal tissues (NT), identified by a pathologist, were obtained from treatment-naïve CRC patients (n = 50) who undertook surgical resection at HMC. Informed consents were obtained from all patients before sample collection. The pathological and clinical characteristics of the study cohort are listed in Table 1.

Tissue specimens were stored in freezing media (10% DMSO, 40% RPMI 1640 media and 50% fetal bovine serum) and used in batches for analyses.

### 2.2. Cell Segregation from Colorectal Tumor Tissues and Normal Colon Tissues

Cells from NT and TT were isolated by mechanical disaggregation, as previously described [10]. Frozen tissues were thawed, washed twice with phosphate-buffered saline (PBS) and mechanically cut into small sections (~2–4 mm). Tissue disaggregation was performed by using gentleMACS^TM^ Dissociator (Miltenyi Biotech, Bergisch Gladbach, Germany) in the absence of proteolytic enzymes. A cell strainer (100 µM) was used to eliminate cell aggregates and debris. The cell suspension was washed with PBS and stained with different antibodies for multi-parametric flow cytometric analyses.

### 2.3. Flow Cytometric Analyses

Cells were re-suspended in 100 µL flow cytometry staining buffer (0.1% sodium azide and 1% FCS in PBS; FACS buffer). FcR Blocking reagent (Miltenyi Biotech) was added for blocking Fc receptors. 7-Aminoactinomycin D (7-AAD) Viability Staining Solution (BioLegend, San Diego, CA, USA) was added to gate live cells. Cell surface antibodies against CD3-BrilliantTM Ultraviolet 496 (BUV496; clone UCHT-1; BD Biosciences, Oxford, UK), CD4-Fluorescein Isothiocyanate (FITC; clone RPA-T4; BD Biosciences), CD8-Phycoerythrin Cyanine 7 (PE/Cy7; clone RPA-T8; BioLegend, San Diego, CA, USA), CD19-Allophycocyanin Cyanine 7 (APC-Cy7; clone HIB19; eBioscience, San Diego, CA, USA), CD56-Phycoerythrin (PE; clone B159; BD Biosciences), PD-1-PE/DazzleTM 594 (clone EH12.2H7; BioLegend), TIM-3-Brilliant Violet 711 (BV711; clone 7D3; BD Biosciences), LAG-3-Brilliant violet 421 (clone T47-530; BD Biosciences), TIGIT-APC (clone MBSA43; eBioscience) and ICOS-Alexa Fluor 700 (AF700; clone C398.4A; BioLegend) were added, and cells were incubated for 30 min at 4 °C. Cells were washed twice and resuspended in FACS buffer. Fluorescence minus one (FMO) control(s) and isotype-matched control(s) were used for data interpretation and validation of antibody staining for phenotypical analyses. Data acquisition was performed on a BD LSRFortessa X-20 flow cytometer running on BD FACSDiva software (BD Biosciences), and the generated data were analyzed on FlowJo V10 software (FlowJo, Ashland, OR, USA).

### 2.4. Analyses Using the Cancer Genome Atlas (TCGA) Colorectal Cancer

Certain genes, based on the protein expression on immune cell infiltrates from our findings, were selected to extract RNA Seq data for these genes for analyses in TCGA CRC dataset. The survival and phenotype data were obtained from TCGA database to plot Kaplan–Meier survival curves and calculate log-rank *p* values using Graphpad Prism 8. Chi-squared χ^2^ test was used to check he statistical significance of the distribution of CRC patients across different disease stages.

### 2.5. Statistical Analyses

Statistical analyses were performed on GraphPad Prism 8 software (GraphPad Software, San Diego, CA, USA). In grouped analyses, One-way Anova test was used to investigate statistical significance. Paired t-tests/Wilcoxon matched-pairs signed rank tests were implemented within groups and Unpaired t-tests/Mann–Whitney between groups, based on Shapiro–Wilk normality test to establish data distribution. In all analyses, a *p* value of <0.05 was considered statistically significant and represented as: *; *p* < 0.05, **; *p* < 0.01 and ***; *p* < 0.001. Presented data show mean ± standard error of the mean (SEM).

## 3. Results

### 3.1. Tumor-Infiltrating Immune Cells in Colorectal Tumors

**Figure 1:**
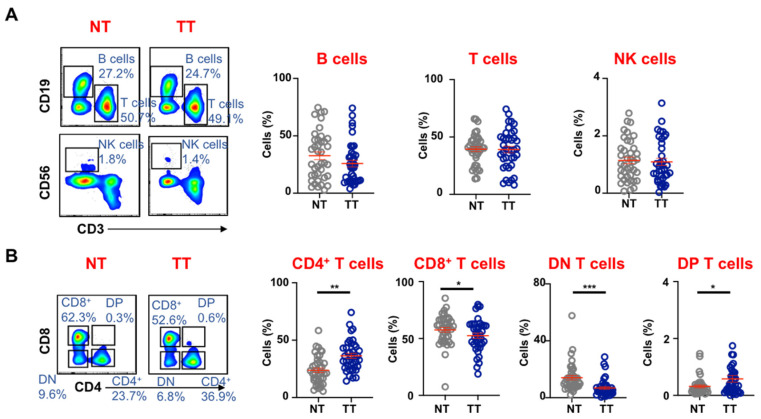
Lymphocyte populations in the colorectal tumor microenvironment. Levels of lymphoid cells were measured in the tumor microenvironment of colorectal cancer (CRC) patients, compared with their levels in normal tissues. Cells were isolated from tumor tissues (TT) and normal tissues (NT) of 50 CRC patients for flow cytometric analyses. CRC patients for flow cytometric analyses. Representative flow cytometric plots and cumulative scatter plots show levels of CD19^+^ B cells, CD3^+^ T cells and CD56^+^ NK cells in NT and TT (**A**). Representative flow cytometric plots and cumulative scatter plots show levels of different CD3^+^ T cell subsets; CD4^+^, CD8^+^, CD4^−^CD8^−^(DN), and CD4^+^CD8^+^ (DP) T cells in NT and TT (**B**). The *p* values are represented as follows; * *p* < 0.05, ** *p* < 0.01, *** *p* < 0.001.

We performed phenotypical characterization of TIICs and compared with immune cells in normal colon tissues of CRC patients (n = 50). We focused on lymphoid cells due to their cardinal roles in anti-tumor immune responses. We found that B cells and T cells are abundantly present in normal colon milieu and in the CRC TME, compared to NK cells (Figure 1A). Importantly, we found that levels of these cells were similar in NT and TT, although B cells showed a trend towards reduced levels in the TME, but the data did not reach statistical significance. NK cells also showed a statistically insignificant, trend towards lower levels in the TME compared to normal tissue.

Next, we focused our investigations on the phenotypical characterization of T cells in normal tissue-infiltrating lymphocytes (NILs) and tumor-infiltrating lymphocytes (TILs) (Figure 1B). We investigated levels of four main subtypes of T cells; CD4^+^, CD8^+^, double negative (CD4^−^CD8^−^; DN) and double positive (CD4^+^CD8^+^; DP) T cells, and found that the majority of T cells in NT and TT comprised of CD4^+^ or CD8^+^ T cells, followed by DN T cells while DP T cells were present at lower levels. Importantly, we found that CD4^+^ T cells and DP T cells were present at significantly higher levels in TT compared to NT (CD4^+^: NT; 23.7 ± 2.0 vs. TT; 36.3 ± 2.2, DP: NT; 0.3 ± 0.1 vs. TT; 0.6 ± 0.1), while CD8^+^ T cells and DN T cells were present at significantly lower levels in TT compared to NT (CD8^+^: NT; 57.6 ± 2.1 vs. TT; 52.4 ± 2.2, DN: NT; 14.0 ± 1.6 vs. TT; 6.8 ± 1.0) (Figure 1B).

### 3.2. Immune Checkpoint Expression on Lymphoid Cell Populations in CRC TME

**Figure 2:**
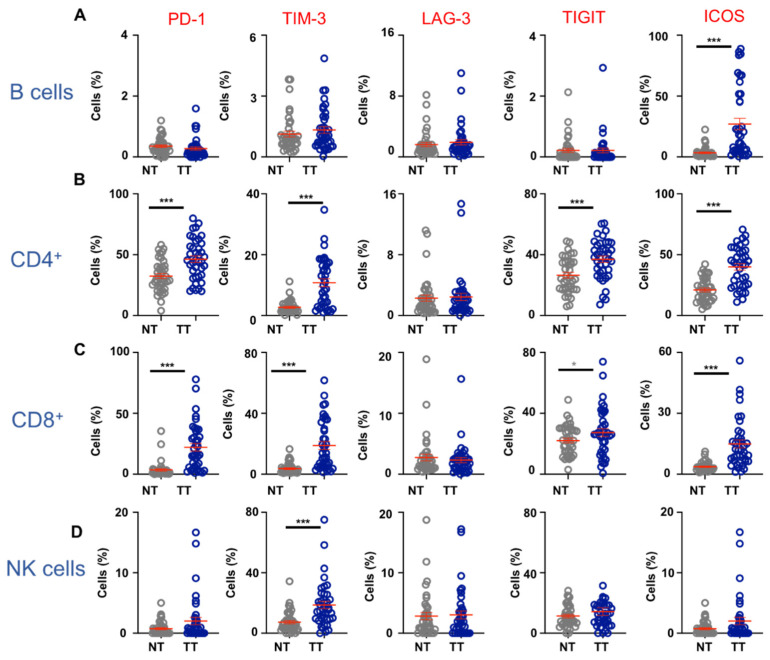
Immune checkpoint expression on different lymphocyte populations in the colorectal tumor microenvironment. We investigated differences in levels of immune checkpoints; PD-1, TIM-3, LAG-3, TIGIT, and ICOS-expressing B cells, CD4^+^ T cells, CD8^+^ T cells and NK cells in NT and TT of 50 CRC patients. Cumulative scatter plots show differences in levels of IC-expressing B cells (**A**), CD4^+^ T cells (**B**), CD8^+^ T cells (**C**), and NK cells (**D**) in NT and TT. The *p* values are represented as follows; * *p* < 0.05, *** *p* < 0.001.

PD-1, TIM-3, LAG-3, TIGIT, and ICOS are identified as important co-inhibitory/stimulatory receptors expressed on immune cells [19]. While expression of these receptors is almost exclusively studied on T cells, their expression levels on other lymphoid cells in the CRC TME remain to be elucidated. Therefore, we investigated expression of these receptors on TIICs from colorectal tumors and compared their expression on cells isolated from colon NT (Figure 2). We found that co-inhibitory molecules PD-1, TIM-3, LAG-3, and TIGIT were expressed on relatively low levels on B cells in NT and TT with some evidence of upregulation on B cells isolated from TT (Figure 2A). In contrast, co-stimulatory receptor ICOS on B cells was expressed at significantly higher levels in TT, compared with NT (NT; 3.4 ± 0.7 vs. TT; 27.1 ± 4.7, Figure 2A).

Tumor-infiltrating CD4^+^ T cells showed significantly elevated expression of PD-1 (NT; 32.5 ± 2.0 vs. TT; 46.3 ± 2.7), TIM-3 (NT; 2.7 ± 0.3 vs. TT; 10.8 ± 1.3) and TIGIT (NT; 26.5 ± 1.8 vs. TT; 37.0 ± 2.1), but not LAG-3 (Figure 2B). Interestingly, ICOS was also significantly overexpressed on CD4^+^ TILs than NILs (NT; 21.0 ± 1.5 vs. TT; 40.0 ± 2.6). CD8^+^ TILs also showed a similar pattern of IC expression as CD4^+^ TILs in CRC TME; PD-1 (NT; 3.5 ± 1.0 vs. TT; 22.0 ± 3.1), TIM-3 (NT; 3.7 ± 0.6 vs. TT; 18.9 ± 2.6), TIGIT (NT; 22.1 ± 1.6 vs. TT; 27.3 ± 2.6) and ICOS (NT; 3.7 ± 0.3 vs. TT; 14.9 ± 2.0) were significantly overexpressed on CD8^+^ TILs compared to cells isolated from NT (Figure 2C).

Next, we investigated IC expression on NK cells from NT and TT of CRC patients. Crucially, we found that of all the ICs investigated, only TIM-3 was expressed at significantly higher levels on NK cells in TT compared to NT (NT; 7.3 ± 1.1 vs. TT; 18.7 ± 2.4, Figure 2D).

### 3.3. Immune Checkpoint Co-Expression on T Cells in the CRC TME

**Figure 3:**
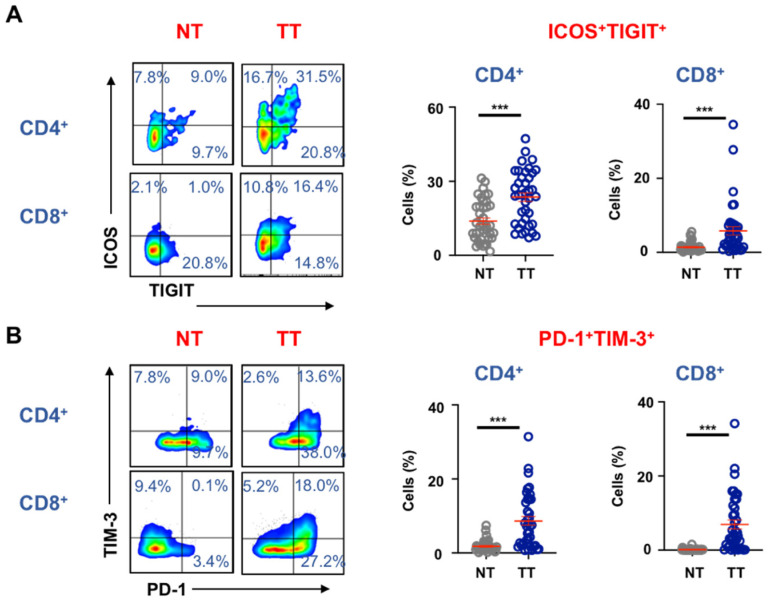
Immune checkpoint co-expression on different T cell subsets in the colorectal tumor microenvironment. We investigated co-expression of TIGIT/ICOS and PD-1/TIM-3 on CD4^+^ T cells, CD8^+^ T cells in TT and NT of 50 CRC patients. Representative flow cytometric plots and cumulative scatter plots show differences in levels of ICOS^−/+^TIGIT^−/+^ (**A**) and PD-1^−/+^TIM-3^−/+^ (**B**) on CD4^+^ and CD8^+^ T cells in NT and TT. The *p* values are represented as follows; *** *p* < 0.001.

Recent studies have shown that multiple ICs can be expressed on TILs and have important implications on their roles in tumor progression. For instance, Yang et al., have recently shown that TIGIT^+^PD-1^+^ T cells are functionally exhausted with impaired ability to induce anti-tumor immune responses [20]. Similarly, PD-1^+^TIM-3^+^ TILs exhibit the most severe exhausted phenotype in the TME [21]. In addition, Di Blasi et al., showed that accumulation of ICOS and TIGIT co-expressing TILs in hepatocellular carcinoma, which were functionally impaired [22]. Therefore, we investigated the levels of ICOS+TIGIT+ and PD-1^+^TIM-3^+^ TILs in CRC tumors compared to NT (Figure 3). We found that both CD4^+^ICOS^+^TIGIT^+^ (NT; 13.9 ± 1.3 vs. TT; 23.6 ± 1.7) and CD8^+^ICOS^+^TIGIT^+^ (NT; 1.4 ± 0.2 vs. TT; 5.8 ± 1.1) cells were present at significantly higher levels in TT compared to NT (Figure 3A). Likewise, we found that CD4^+^PD-1^+^TIM-3^+^ (NT; 1.8 ± 0.2 vs. TT; 8.7 ± 1.1) and CD8^+^PD-1^+^TIM-3^+^ (NT; 0.2 ± 0.1 vs. TT; 10.4 ± 1.9) cells were also present at significantly higher levels in TT compared to NT (Figure 3B).

### 3.4. Tumor-Infiltrating Immune Cells in Advanced and Early Stage CRC Patients

**Figure 4:**
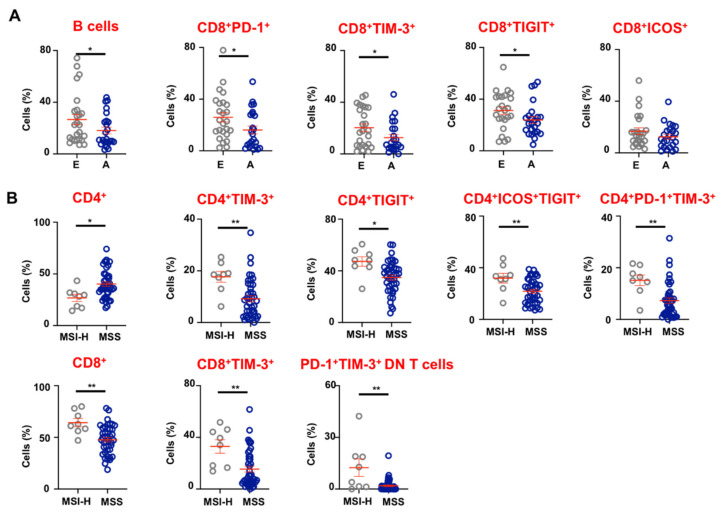
Levels of tumor-infiltrating lymphoid cells in CRC patients with different disease stages and microsatellite instability-high (MSI-H) versus microsatellite stable (MSS) tumors. We divided the 50 patients into two groups; early Scheme 25. and advanced stage (III and IV, n = 25, and investigated differences in levels of tumor-infiltrating lymphocytes (TILs) between the two groups. Cumulative scatter plots show differences in levels of B cells, CD8^+^PD-1^+^, CD8^+^TIM-3^+^, CD8^+^TIGIT^+^, and CD8^+^ICOS^+^ T cells in CRC patients with advanced stage compared to early stage (**A**). We also divided patients into two groups based on expression of MMR proteins to identify patients with MSI-H (n = 8) and MSS (n = 42) tumors, and investigated differences in levels of TILs between the two groups. Cumulative scatter plots show differences in levels of CD4^+^, CD4^+^TIM-3^+^, CD4^+^TIGIT^+^, CD4^+^ICOS^+^TIGIT^+^, and CD4^+^PD-1^+^TIM-3^+^ T cells, and CD8^+^, CD8^+^TIM-3^+^ and CD4^+^, PD-1^+^TIM-3^+^ DN T cells, in CRC patients with MSI-H compared to MSS tumors (**B**). The *p* values are represented as follows; * *p* < 0.05, ** *p* < 0.01.

To elucidate the plausible roles of various IC-expressing lymphoid cells in the TME with the progression of CRC, we compared the levels of different IC-expressing cellular populations between patients with advanced (III and IV, n = 25) and early (I and II, n = 25) stages. First, we investigated differences in levels of B cells, T cells and NK cells between patients with advanced and early stage CRC. We found that levels of tumor-infiltrating B cells were present at significantly lower levels in patients with advanced stage disease (A; 18.1 ± 2.6 vs. E; 26.6 ± 4.0, Figure 4A). However, other cellular populations including different T cell subsets did not show any statistically significant differences (data not shown).

Next, we compared the levels of tumor-infiltrating B cells, T cells and their subsets, and NK cells expressing PD-1, TIM-3, LAG-3, TIGIT, and ICOS between patients with advanced and early stage CRC. Interestingly, we found significantly lower levels of PD-1 in advanced stage CRC (A;16.2 ± 3.0 vs. E; 26.0 ± 3.6), TIM-3 (A; 12.6 ± 2.4 vs. E; 20.3 ± 3.0) and TIGIT (A; 23.9 ± 2.6 vs. E; 31.2 ± 3.0)-expressing CD8^+^ T cells in the TME compared to CRC patients with early stage disease. Moreover, CD8^+^ICOS^+^ TILs also showed a trend of reduction in patients with advanced stage disease, but the data did not reach statistical significance. Other IC-expressing cellular populations did not show any differences between patients with advanced and early stage CRC (data not shown).

### 3.5. CRC Patients with dMMR/MSI-H Tumors Have Higher Levels of CD8^+^ TILs and Exhibit Elevated Expression of Immune Checkpoints

Eight patients out of our total patient cohort (n = 50) exhibited MSI-H, as confirmed by pathological assessment of TT (loss of expression of MMR proteins; MLH1, MSH2, MSH6 and PMS2 by immunohistochemistry). We compared the levels of different tumor-infiltrating lymphoid cells and IC expression, between patients exhibiting MSI-H versus MMR proficient/MSS tumors (Figure 4B). We found that patients with MSI-H tumors had significantly lower levels of CD4^+^ TILs compared to MSS tumors (MSI-H; 26.8 ± 3.4 vs. MSS; 40.2 ± 2.1). Moreover, CD4^+^ TILs from patients with MSI-H tumors showed significantly higher expression of TIM-3 (MSI-H; 17.7 ± 2.1 vs. MSS; 9.2 ± 1.2), TIGIT (MSI-H; 47.3 ± 3.3 vs. MSS; 34.8 ± 1.9), and co-expression of PD-1/TIM-3 (MSI-H; 15.1 ± 2.1 vs. MSS; 7.3 ± 1.1) and ICOS/TIGIT (MSI-H; 32.0 ± 3.7 vs. MSS; 21.8 ± 1.5), compared to MSS tumors. In contrast, MSI-H tumors showed significantly higher levels of CD8^+^ TILs (MSI-H; 64.3 ± 4.0 vs. MSS; 47.7 ± 2.1), which expressed TIM-3 (MSI-H; 33.0 ± 5.2 vs. MSS; 15.4 ± 2.2) at significantly higher levels than MSS tumors. In addition, PD-1 and TIM-3 co-expressing DN T cells were also present at significantly higher levels in MSI-H tumors compared to MSS tumors (MSI-H; 16.6 ± 6.8 vs. MSS; 2.5 ± 0.7). Of note, we did not find any statistically significant differences between levels of overall and IC-expressing B cell, CD3^+^ T cell and NK cell populations (data not shown).

### 3.6. TCGA Datasets Showed Higher Expression of Lymphoid-Related and IC Genes in Early Stages of CRC

**Figure 5:**
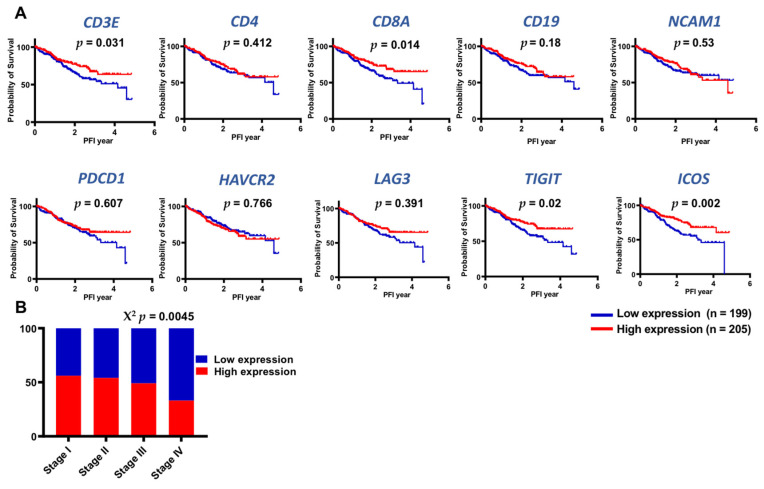
Evaluation of patient progression-free interval data for selected genes from TGCA COADREAD dataset. Kaplan-Meier curves for progression-free interval (PFI) were compared between patients with high (n = 205) and low (n = 199) gene expression of *CD3E*, *CD4*, *CD8A*, *CD19*, *NCAM1*, *PDCD1*, *HAVCR2*, *LAG3*, *TIGIT*, and *ICOS* (**A**). Distribution of patients with high and low gene expression of selected genes across different stages using Chi-squared (χ^2^) test (Stages I, II, III, and IV) (**B**).

Our data showed similar levels of B cells, T cells and NK cells in the CRC TME compared to NT, but these lymphoid cell populations show elevated expression of selective ICs in the TME. Importantly, we found that patients with advanced stage disease have lower levels of tumor-infiltrating B cells and IC-expressing CD8^+^ T cells. To find out the plausible clinical significance of these results, we investigated associations between elevated expression levels of *CD3E*, *CD4*, *CD8A*, *CD19*, *NCAM1* (gene for CD56), *PDCD1* (gene for PD-1), *HAVCR2* (gene for TIM-3), *LAG3*, *TIGIT,* and *ICOS* genes with clinical parameters including disease-specific survival (DSS), overall survival (OS), and progression-free interval (PFI) from TCGA datasets of CRC patients (Figure 5). We divided patients into two groups based on median expression levels of these genes, low expression and high expression, and investigated associations between individual gene expression and patient survival. We did not find any significant association between elevated expression of these genes with OS and DSS (data not shown). Survival analyses using Kaplan–Meier and log-rank test showed that low expression levels of genes including *CD3E* (*p* = 0.031), *CD8A* (*p* = 0.014), *TIGIT* (*p* = 0.020), and *ICOS* (*p* = 0.002) were significantly associated with poorer PFI (Figure 5A). Next, we investigated associations between combined elevated expression levels of *CD3E*, *CD4*, *CD8A*, *CD19*, *NCAM1*, *PDCD1*, *HAVCR2*, *LAG3*, *TIGIT,* and *ICOS* genes with disease staging (Figure 5B). We found that patients with advanced stages (stages III and IV) showed decreased expression levels of these genes compared to patients with early stage disease (stages I and II) (χ^2^ *p* = 0.045, Figure 5B). Taken together, the TCGA analyses showed that advanced stage CRC patients have reduced levels of lymphoid and IC genes but importantly, lower expression levels of *CD3E*, *CD8A*, *TIGIT,* and *ICOS* were associated with poorer PFI.

## 4. Discussion

TIICs are vital indicators of anti-tumor immune responses and can ultimately affect disease prognosis in multiple cancers including CRC [23]. Conclusive evidence showed that Th-1-polarized CD8^+^ T cells and presence of CD45RO^+^ memory T cells can influence tumor progression and metastasis [5,24]. Thus, introduction of “immunoscore” based on the levels of CD3^+^ and CD45RO^+^, CD3^+^ and CD8^+^, or CD8^+^ and CD45RO^+^ in the core tumor (CT) and at the invasive margin (IM) by Galon et al., showed superior prognostic ability in CRC patients [25]. Moreover, within the TME, different immune cell populations perform their specialized functions such as NK cells involved in lysis of tumor cells, which lose MHC class I expression [26], and B cells, which can modulate immune responses via various mechanisms, including inhibition of T cell responses [27]. Importantly, ICs have emerged as important molecules, which can affect the functionality of immune cells, as evident by activation/hyperactivity of T cells or T cell exhaustion exhibited by IC-expressing T cells.

Shimabukuro-Vornhagen et al., have previously shown elevated levels of tumor-infiltrating B cells compared to circulation in CRC patients [28]. Importantly, the authors showed that these tumor-infiltrating B cells were highly activated and showed elevated levels of memory B cells in the TME and in circulation of CRC patients compared with circulation of healthy donors [28]. However, the authors did not compare the levels with tissue resident B cells in normal colon tissue. Our data showed that the levels of overall B cells were similar between tumor and normal tissue, but express significantly higher levels of co-stimulatory IC, ICOS in the TME. These data show that B cells within the TME may support T cell activation. Of note, an important subset of B cells recognized as B regulatory cells (Bregs) has emerged as a potent immunosuppressive cell type, which can attenuate anti-tumor immune responses [27]. Further investigations are warranted to identify the levels of Bregs within CRC TME to disclose the balance between different T cell stimulating or inhibiting B cell subsets within the CRC TME.

Elevated T cell infiltration in CRC tumors is concomitant with improved survival [29]. Sherwood et al., reported that TCR repertoire analyses of T cells in CRC TME compared to normal tissues showed wider diversity than variation of T-cell clones in healthy mucosa [30]. Our data provide important insights into the distribution of T cells within the TME; subsets of CD4^+^ T cells and DP T cells have been shown to have immunosuppressive roles in CRC [31,32,33], while DN T cells may also exhibit immunoregulatory potential [34] but CD8^+^ T cells within TME show potent anti-tumor activity evident from granzyme B expression [35]. In addition, infiltration of NK cells alongside CD8^+^ T cells in the TME, has been shown to be associated with improved prognosis in CRC patients [36]. Ndhlovu et al., reported that NK-cell responses are negatively regulated by interactions of TIM-3-expressing NK cells with cognate ligands of TIM-3 [37]. Overall, the balance of the different T cell subsets in favor of lower levels of CD8^+^ T cells compared to CD4^+^ T cells, and TIM-3^+^ NK cells, which can further dampen T cell responses within CRC TME, highlighting an immune tolerant environment to aid tumor progression.

Immune checkpoints on T cells influence response to TCR ligation by cognate antigen and can affect downstream signaling pathways for their activation, survival, and function [38]. Importantly, ICs are also over-expressed on exhausted T cells, characterized by their inability to produce effector cytokines [39]. PD-1, TIM-3, and TIGIT are negative regulators of T cell activation, which suppress their activation, proliferation and cytokine production upon interactions with their ligands [19]. In contrast, ICOS serves as a co-stimulatory molecule with opposing effects on T cell activation, proliferation and functionality [19]. The prognostic significance of ICs in CRC has been explored previously [11,40]. We identified that only CD8^+^ T cells expressing different ICs were found at lower levels in CRC patients with advanced stage disease. Furthermore, we showed that some of these ICs are co-expressed on tumor-infiltrating T cell subsets in CRC patients. Co-expression of multiple ICs can have important implications on resistance mechanisms to immunotherapy by unique or overlapping IC pathways, which may assist tumor progression [41,42].

Chromosomal instability is identified as a prerequisite for the development of majority of CRC and around 12–15% of all CRC tumors exhibit dMMR, which is characterized by MSI [13]. MSI-H CRC tumors have been previously reported to have high immune cell infiltration, which include CD8^+^, Th1 TILs, and macrophages [43,44]. Moreover, associations between elevated levels of tumor-infiltrating T cells and MSI-H in CRC patients have also been reported [45]. These evidences suggest that CRC patients with MSI-H could respond better to immunotherapy treatments and also have better disease prognosis. However, disease prognosis remains worse in majority of CRC patients with advanced stage disease, devoid of MMR status, implying the presence of other mechanisms driving tumor progression or hampering response to therapy in such patients. Importantly, Llosa et al., reported that MSI-H tumors could potentially offset the Th1/CTL balance in the CRC TME by upregulation of multiple ICs [46].

To translate the clinical relevance of our findings, we investigated associations between protein expression of genes under scrutiny in our study with patient survival data from TCGA datasets. We found that high expression of genes related to CD3^+^ and CD8^+^ T cells, *TIGIT* and *ICOS* showed an association with a longer PFI. Moreover, patients with advanced stage disease showed lower levels of these genes. These finding are in agreement with our data that CRC patients with advanced stage disease showed lower levels of IC-expressing CD8^+^ T cells in the TME. In contrast, it has been reported that the elevated mRNA expression levels of *CTLA-4*, *TIM-3*, *TIGIT*, and *VISTA* among others were elevated in the TME of CRC patients with advanced stage disease, compared with early stages [40]. Kitsou et al., have recently reported associations between elevated *PD-1* and *TIGIT* gene expression with better overall survival, and reported associations between TIL load and *HAVCR2*, *LAG*-3 and *TIGIT* in CRC patients [11]. Chen et al. also reported elevated expression of PD-L1, TIL load and mutation burden in low-risk CRC patients [47]. Importantly, the consensus molecular subtypes (CMS) classified CRC subtypes into four groups based on genetic modifications and tumor immune profiling; CMS1 (MSI-H, immune activated), CMS2 (activated WNT and MYC signaling), CMS3 (dysregulation of metabolic pathways) and CMS4 (immune inflamed, TGF-β activation) [48]. While, CMS1 tumors exhibit potent anti-tumor activity, transcription of genes encoding ICs such as *PDCD1*, *CD274*, *CTLA4,* and *LAG3* may aid tumor immune escape in these tumors [49].

## 5. Conclusions

**Figure 6:**
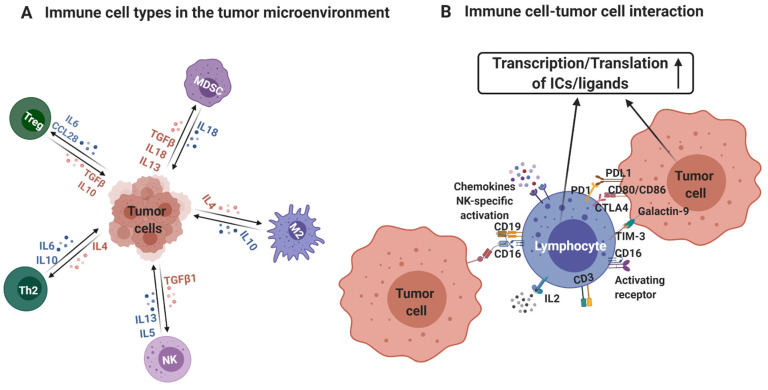
Schematic representation of the upregulation of immune checkpoints/ligands within the tumor microenvironment. Figure shows the immune cell types within the tumor microenvironment (TME) and corresponding cytokines/chemokines for their differentiation and trafficking to the TME (**A**). Immune cells (B cells/NK cells/T cells) express multiple immune checkpoints such as PD-1, CTLA-4, and TIM-3, while tumor cells express PD-L1, CD80/86, and Galectin-9. These immune checkpoints bind with corresponding ligands leading to their transcriptional and translational upregulation through various post-transcriptional/translational modifications (**B**).

Previously reported findings from gene expression, combined with our data of protein expression of different IC genes on various immune cells present in the TME of CRC patients provide important insights in the timely field of cancer immunotherapy. We found that multiple ICs are differentially upregulated/co-expressed on various immune cells in the TME and decreased levels of B cells and selective IC-expressing CD8^+^ TILs are associated with tumor progression. Importantly MSI-H tumors could show favorable prognosis/improved response to cancer immunotherapy due to elevated levels of CD8^+^ TILs and elevated levels of IC-expressing CD4^+^ TILs compared to MSS tumors. These data show that IC pathways can have significant implications on the prognosis and therapy response in CRC patients and highlight important IC molecules, in addition to PD-1, which may be targeted for therapeutic benefits. Figure 6 provides an overview of the presence of multiple lymphoid cell populations, and the expression and transcriptional regulation of ICs and their ligands within the CRC TME. Investigating the functional activity of the different lymphoid cell populations and performing functional annotations for these genes would add to the importance of these data, ultimately for uncovering means of resistance to cancer immunotherapy.

## Figures and Tables

**Table 1:** Clinical and pathological features of study cohort.

	CRC Patients
**Number**	50
**Age** (range)	59 ^†^ (18–83)
**Gender** (Male:Female)	32:18
**TNM stage**	
I	4
II	21
III	16
IV	9
**Tumor histological grade**	
G2 (Moderately differentiated)	45
G3 (Poorly differentiated)	5
**dMMR/MSI-H**	8

CRC; colorectal cancer; dMMR/MSI-H; Mismatch-repair deficiency/Microsatellite instability-high; ^†^ Median age. Bold represents main text/subheading.

## Data Availability

The data presented in this study are available on request from the corresponding author.
